# Carotid body paraganglioma metastatic to spine causing cord compression: a case report

**DOI:** 10.1186/s13000-023-01314-y

**Published:** 2023-02-24

**Authors:** Jazmyne N. Tabb, Jared A. Maas, Bhargav P. Earla, Kenneth B. Fallon, Andrew M. McDonald, Michael C. Dobelbower

**Affiliations:** 1 University of South Alabama College of Medicine, Mobile, USA; 2 Department of Radiation Oncology, University of Alabama at Birmingham, Birmingham, USA; 3 UAB Heersink School of Medicine, University of Alabama at Birmingham, Birmingham, USA; 4 Department of Pathology, University of Alabama at Birmingham, Birmingham, USA

**Keywords:** Carotid body tumor, Paraganglioma, Metastatic, Cord compression, Radiotherapy

## Abstract

**Background:**

Carotid body tumors (CBTs) are rare neuroendocrine neoplasms arising near the carotid bifurcation with a reported incidence of 1 to 2 cases in 100,000 patients. Most CBTs are sporadic, benign, slow-growing, and non-secreting, but untreated CBTs can grow locally to compress the nearby blood vessels, esophagus, and airway. Regional metastases can occur in 5% to 10% of cases, but distant metastases are exceedingly rare, occurring in roughly 1–2% of cases. As such, the optimal treatment for metastatic CBTs is not well-defined. We report a rare case of a patient with CBT distant metastases causing spinal cord compression.

**Case presentation:**

A 40-year-old African American female presented with a right neck mass, headaches, vertigo, tinnitus, hoarseness, and dysphagia. Imaging demonstrated a Shamblin II right neck mass; subsequent transcervical resection and pathology showed a carotid body paraganglioma. The patient recurred locally near the carotid bifurcation, so she underwent Stereotactic Body Radiation Therapy to the recurrent right neck disease. She later re-presented with new onset bilateral lower extremity weakness, dysmetria, and numbness. She was found to have metastatic disease to the thoracic spine causing spinal cord compression. She underwent laminectomy, tumor resection, and posterior fixation followed by adjuvant radiation therapy. She was started on systemic therapy with sunitinib. She eventually progressed with metastatic disease to the right iliac bone, which was treated with palliative radiotherapy. Second line systemic therapy with capecitabine and temozolomide was started. At last follow up, the patient was asymptomatic with stable persistent disease.

**Conclusions:**

Paragangliomas often exhibit a prolonged interval to the development of progression; locoregional recurrences or rare distant metastases have been reported to occur as many as 20 years from diagnosis. The natural course of CBTs in other cases as well as the present case call into question the idea that CBTs are truly benign; instead CBTs may be indolent tumors with metastatic potential. Treatment choices for CBTs include surgical resection, radiation therapy, and systemic therapy, though the optimal treatment regimen for metastatic CBTs is not well-defined. A more advanced understanding of CBT pathophysiology, disease classification, risk stratification, and treatment options is needed to improve outcomes for patients.

## Background

Carotid body tumors (CBTs), generally referred to as paragangliomas, chemodectomas, and glomus tumors, are rare, highly vascular neuroendocrine neoplasms. They arise near the carotid bifurcation within glomus bodies (paraganglia) and are derived from the embryonic neural crest [1].

Most paragangliomas arise in the adrenal gland with only 3% of extra-renal tumors manifesting in the head and neck [2]. Of these head and neck paragangliomas, CBTs are the most common [3]. A small percentage of these tumors are associated with mutations in succinate dehydrogenase genes. Most CBTs are sporadic and present between the ages of 40 and 70 [2, 4]. Sporadic CBTs are usually benign, slow growing, and biochemically silent resulting in patients presenting with a painless anterior neck mass or as an incidental finding on radiographic imaging [4]. If left undetected, patients may present with compressive symptoms including fullness, pain, dysphagia, odynophagia, hoarseness, and stridor. Some catecholamine-secreting tumors can cause autonomic dysfunction along with these compressive symptoms. Without intervention, distant metastases (though rare) and local invasion can be life threatening [5].

Surgical resection is the treatment of choice for CBTs. The Shamblin classification system uses the tumor’s size and association with the carotid vessels to assess the possibility of surgical resection and associated mortality. Based on this relationship and risk, tumors are classified into 3 groups [6]. Group 1 tumors are localized and do not involve the carotid vessels, allowing for resection. Group 2 lesions partially surround the vessels and are more adherent to the adventitia, but still are capable of resection, and group 3 lesions encircle the carotid vessels and invade the media, excluding sub-adventitious surgical resection as a treatment option [6]. Preoperative adrenergic blockade should be considered when making treatment plans, particularly for secreting tumors, and due to the high vascularity of these tumors, pre-operative embolization is usually necessary [7]. Radiation therapy has been shown to be an effective treatment modality for unresectable tumors, poor surgical candidates, and isolated metastases including to the bone [8].

Only about 5–10% of incident cases of CBT present with regional metastasis and distant disease is very rare [9]. Over a 29 year period between 1981 and 2009, there were only 14 cases of histopathologically confirmed CBT with distant metastases out of 97 cases of malignant CBT reported in the medical literature; this suggests a distant metastasis rate of roughly 1–2% in these rare tumors [8]. Optimal treatment regimens for advanced metastatic CBTs are not yet well defined but currently include modalities such as radiation therapy, systemic therapy, and repeat surgical resection [8]. Here we report a case of a 40-year-old female with malignant CBT with distant metastases and treatment including surgical resection, radiation therapy, and systemic therapy.

## Case presentation

**Fig. 1:**
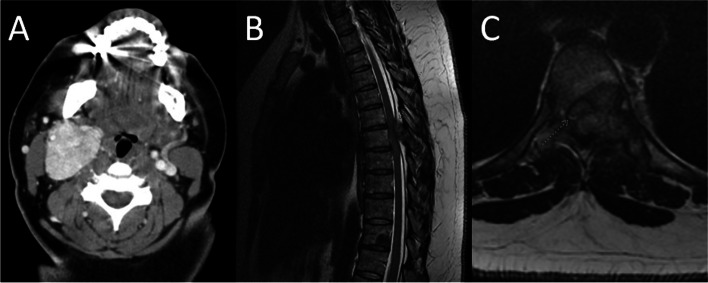
**A** shows an axial CT neck with contrast. There is a 6.1 × 4.0 × 4.1 cm contrast-enhancing right neck mass in the right carotid space centered at C3. The mass partially surrounds the external and internal carotid arteries consistent with a Shamblin group 2 lesion. **B** and **C** show a T2 weighted MR thoracic spine using Fast Recovery Fast Spin Echo (FRFSE). **B** depicts a sagittal view where the arrow indicates posterior to anterior spinal cord compression at the level of T7. There is a sharply circumscribed lesion in the right posterolateral aspect of the T11 vertebral body. **C** depicts an axial view with an expansile mass in the left posterolateral vertebral body, pedicle, laminae, and transverse process of T7. The T7 lesion expands the left posterior epidural compartment compressing the spinal cord anterolaterally towards the right. The arrow points to a small amount of visible cerebrospinal fluid indicating Bilsky grade 2 cord compression

A 40-year-old African American female presented in 2014 with complaints of a right neck mass that was first appreciated 9 months earlier. The appearance of the neck mass coincided with a constellation of symptoms: frequent severe headaches, periods of lightheadedness, vertigo and tinnitus, hoarseness, dysphagia, blurred vision and tearing of the right eye. On physical examination, a non-pulsatile, non-tender mass was palpated, deep to the right sternocleidomastoid muscle. The patient underwent CT neck imaging, which revealed a 6.1 × 4.0 × 4.1 cm right neck mass extending to the base of the skull and encompassing the internal and external carotid artery (Fig. 1A). Upon further evaluation, the tumor was felt to be a Shamblin II lesion because there was some carotid arterial attachment, but the tumor did not entirely encase the carotid arteries, so the tumor was deemed as reasonable for resection. A cerebral arteriogram showed no intracranial arterial vascular abnormality, so an extracranial embolization was performed. Transcervical resection of the right carotid body tumor was performed the following day, and pathology showed a paraganglioma measuring 4.9 cm with areas of infarct and multiple vessels with intravascular thrombi, consistent with prior embolization procedure. The transcervical resection of the right carotid body paraganglioma was a gross total resection, but a microscopic surgical margin assessment on the surgical pathology was not performed. Following surgery, the patient continued to remain symptomatic with headaches, right neck and ear soreness, Horner’s syndrome symptoms, and right vocal cord paralysis. She received right vocal fold injection medialization. Over time, the patient reported improvement in most of her symptoms.

**Fig. 2:**
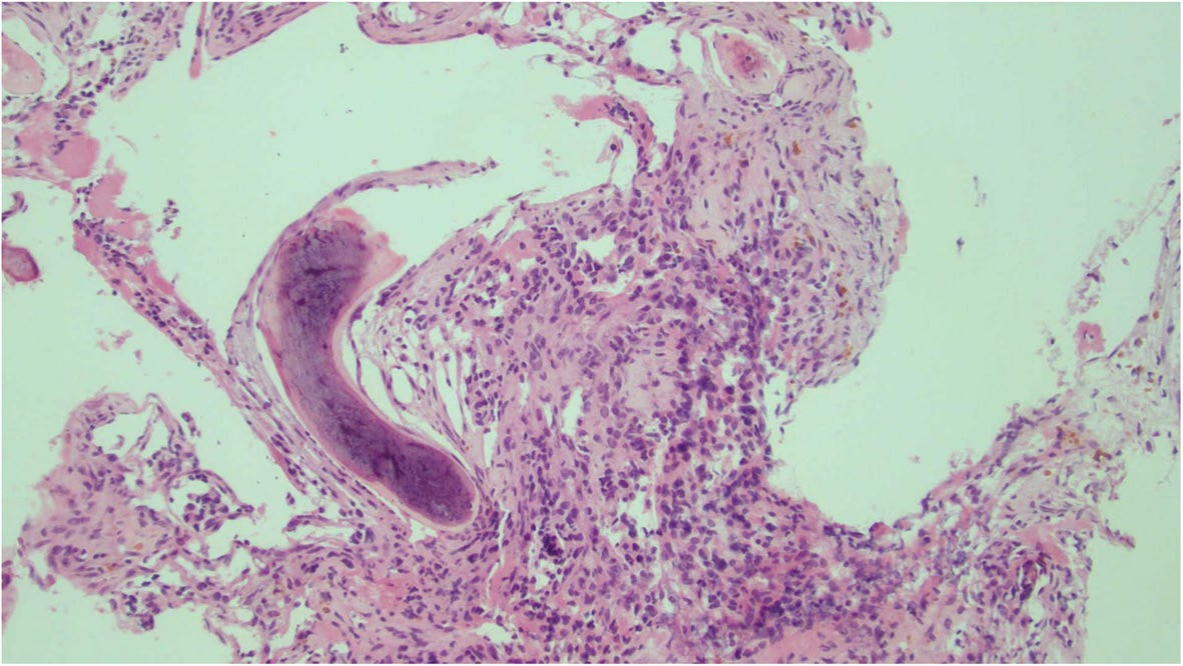
Hematoxylin and eosin staining of the resected thoracic spine tumor shows metastatic paraganglioma characterized by neuroendocrine cells which occupy the marrow spaces of cancellous bone. The semicircular, hematoxylinophilic curved structure at left represents one of the distorted, calcific bone trabeculae. Occasional foci of hemosiderin are present, consistent with chronic hemorrhage. Immunohistochemical stains of the tumor cells are strongly positive for both synaptophysin and chromogranin, and negative for pancytokeratin and Cam5.2. The Ki-67 proliferation index is elevated at about 20%. The staining pattern is consistent with paraganglioma

The patient was routinely followed in office and via imaging. MRI in 2017 showed a small mass measuring about 1 cm in the vicinity of the carotid bifurcation, but it was unclear if this was recurrent disease, so the decision was made to continue to observe the mass. In 2018, a subsequent MRI showed interval enlargement of the mass from 1.4 × 1 × 1.2 cm to 2.5 × 1.7 × 2.4 cm. Due to the disease progression, the patient received Stereotactic Body Radiation Therapy of 25 Gy in 5 fractions to the presumed recurrent right paraganglioma in 2018. For the next two years, the patient’s imaging and clinical symptoms remained stable. In 2021, the patient re-presented with six months of new onset thoracic radiculopathy and weight loss, and one month of progressive bilateral lower extremity weakness, dysmetria, and paresthesias and numbness from the umbilicus down. Upon physical examination, the patient's strength was 4 + /5 in her bilateral lower extremities, she had decreased sensation to light touch and pin-prick from her naval to her distal bilateral lower extremities, she had intact sensation to light perineal touch, her bilateral patellar reflexes were 2 + , and her bilateral ankle reflexes had single beat clonus. She had a mildly ataxic gait with dysmetria on heel to toe walk and heel walk. While her finger to nose testing was intact, her heel to shin testing revealed dysmetria. During rectal examination, the patient demonstrated brisk voluntary anal contraction. MRI of the T spine showed a spinal mass arising from the posterior elements at T6-T7 with at least Bilsky grade 2 posterior to anterior cord compression as well as a mass in the T11 vertebral body (Fig. 1B and C). This compressive pathology localized to the patient’s acute symptoms, and it was determined that she would require surgical intervention. She underwent T6-T7 laminectomy, T5-T7 tumor resection, and T5-T9 posterior fixation in 2021 with pathology showing metastatic paraganglioma (Fig. 2). She subsequently completed radiation therapy of 30 Gy in 10 fractions to the thoracic spine from T4 to T11 to encompass both the surgical field as well as the T11 metastasis in 2021.

Following completion of radiation therapy to the thoracic spine, restaging DOTATATE PET in mid-2021 showed multifocal uptake in the right carotid body surgical bed as well as uptake in T4, T11, and the right iliac. Genetic evaluation showed no evidence of pathogenic mutations. The patient was evaluated for 131Iodine-Iobenguane but she was not felt to be a candidate. The patient was started on systemic therapy with sunitinib complicated by mucositis. The patient’s sunitinib dose was decreased to help alleviate the mucositis. Interval DOTATATE PET in late 2021 showed decreased size and uptake of the right neck masses and osseous metastases consistent with treatment response. The patient reported 2–3 months of dull, burning right hip pain. An MRI was obtained showing a well marginated lesion within the right iliac bone in the area of PET avidity consistent with metastatic disease. The right iliac lesion was treated with 8 Gy of radiation therapy in a single fraction for right hip pain attributed to bony metastatic disease. DOTATATE PET in mid 2022 showed two areas of focal uptake in the right carotid body surgical bed, small volume mild uptake in the C5 and T11 vertebral bodies and the right iliac, and a left adrenal nodule without PET uptake. Therefore, the patient’s systemic therapy was switched to capecitabine and temozolomide. At the time of her last follow up in 2022—a total of 13 months since completing thoracic spinal surgery and radiotherapy and 4 months since completing palliative right hip radiotherapy—the patient was ambulatory, her previously reported neurologic deficits had resolved, and she had no right hip pain. Upon physical examination at last follow up, the patient's strength was 5/5 throughout, her sensation was normal to light touch, and her reflexes were 2 + throughout. She ambulated independently with intact heel to toe walking, heel walking, and toe walking.

## Discussion and conclusions

Paragangliomas are a rare type of catecholamine-secreting neuroendocrine tumors that originate from neural crest cells [10]. Paragangliomas can be classified as sympathetic or parasympathetic; sympathetic paragangliomas arise from chromaffin cells of paraganglia along the sympathetic chain and parasympathetic paragangliomas arise from the glomera that are distributed along parasympathetic nerves in the head, neck, and upper mediastinum [11]. Paragangliomas located at the carotid bifurcation are known as carotid body tumors, and are extremely rare with a reported incidence of 1–2/100,000. They account for 0.012% of all body tumors and 0.5% of head and neck tumors [12–16].

Sympathetic paragangliomas are usually clinically functional, with symptoms including paroxysmal or sustained hypertension, anxiety, and recurring episodes of headache, sweating, and palpitations [11]. Parasympathetic paragangliomas are usually benign, non-functional, and present as space-occupying lesions causing symptoms such as pain, hoarseness, and dysphagia [11]. Most CBTs are sporadic, but a small percentage of CBTs are familial. Sporadic CBTs are usually benign, slow growing, and biochemically silent [2, 4]. The patient described in the case above presented with headaches, lightheadedness, vertigo and tinnitus, hoarseness, dysphagia, blurred vision of the right eye, and no known familial history, most indicative of a sporadic, benign, space-occupying lesion.

Paragangliomas generally behave like benign tumors with the majority of recurrences occurring locally [11]. Paragangliomas often exhibit a prolonged interval to the development of recurrence or metastasis, with recurrences and/or distant metastasis being reported to occur 0–20 years from diagnosis [11, 17–21]. An estimated 10% of paraganglioma tumors will demonstrate malignant potential, spreading to regional lymph nodes, bone, lung, and liver [11, 19]. Despite distant metastasis being a rare occurrence, the patient described in the case above demonstrated multiple vertebral metastases at T6-T7 and T11, resulting in a Bilsky grade 2 posterior to anterior cord compression. The prognostic outcome of malignant paragangliomas is unfavorable with a 5-year mortality of less than 50% [11]. Characteristics of CBTs—including their metastatic potential and prolonged intervals between definitive treatment and recurrence as in the present case—suggest that CBTs are probably indolent and slow to metastasize, rather than being truly benign.

Treatment choices for CBTs include conservative management, radiation therapy, surgical resection, or a combination of these, but the optimal treatment regimen for advanced metastatic CBTs is not well defined [8]. The patient in the present case had improved neurologic symptoms following a multi-modality treatment regimen including surgery, radiation, and systemic therapy.

Surgical resection is the current treatment of choice for CBTs with high reported rates of local control (94–100%) [22]. Radiation therapy has historically been reserved for unresectable disease, poor surgical candidates, and metastasis to bone [6, 8]. Because of CBTs high vascularity, preoperative embolization has widely been utilized [23]. Two meta-analyses found a statistically significant lower blood loss and shorter duration of operation in patients who underwent preoperative embolization than patients without it [24, 25], however another study did not demonstrate any operative or postoperative advantage of preoperative embolization in patients scheduled for CBT surgery [23]. Preoperative embolization was effective in our patient, as she experienced none of the common postoperative complications including cranial nerve injury or stroke.

Radiation therapy has traditionally served as a second line intervention in the treatment of CBTs, but conventional radiotherapy with fractionated external beam radiotherapy (EBRT) has more recently been examined as a primary treatment in multiple studies. One study reported on 13 patients with CBTs, 10 of which underwent definitive EBRT. Local control was achieved in all patients [26]. A meta-analysis reported the results of 127 patients with CBTs treated with radiotherapy. When comparing radiotherapy with patients who underwent surgical resection, there were no significant differences observed in tumor control or mortality, and radiation conferred a lower risk of major complications compared to surgery alone (OR:1.4; 95% CI:0.66–2.25 vs OR:0; 95% CI:0–0; *p*= 0.047) [27]. The patient in the case above received primary surgical resection, radiosurgery for local recurrence, surgical resection and adjuvant radiation to the thoracic spine, and palliative radiotherapy to the right iliac bone. At the time of last follow up, the patient has PET evidence of recurrence in the right carotid body bed suggesting treatment failure. There is indeterminate low-level activity in the treated T11 and right iliac lesions as well as in the C5 vertebral body, which was not treated. Serial PET imaging suggested that the patient had an initial clinical complete response to sunitinib 5 months following thoracic radiotherapy, but then the patient progressed to stable disease 10 months following thoracic radiotherapy. Crucially, the combination of surgical spinal decompression and adjuvant radiotherapy reversed the patient’s symptomatic spinal cord compression such that she is asymptomatic and fully functional at the time of last follow up.

Current studies support systemic therapy options for paragangliomas with distant metastases including octreotide or lanreotide; sunitinib; temozolomide; capecitabine; combination chemotherapy with cyclophosphamide, vincristine, and dacarbazine; or radionuclide therapy with 131Iodine or 177Lutetium-dotatate [28–32]. The patient in this case received sunitinib, followed by capecitabine and temozolomide. She was not a candidate for 131Iodine Iobenguane.

Risk stratification and investigation into the role of the various treatments for CBT is needed in order to create a protocol that will improve outcomes for patients. Distant metastases is a rare event in CBT but can occur many years after primary treatment, so while routine systemic imaging is not currently recommended for all patients, long term clinical follow up and attention to patient-reported symptoms may help to identify distant metastasis sooner.

## Data Availability

Data sharing is not applicable to this article as no datasets were generated or analyzed during the current study.

## Abbreviations

CBT: Carotid Body Tumor
EBRT: External Beam Radiotherapy
OR: Odds Ratio
CI: Confidence Interval
FRFSE: Fast Recovery Fast Spin Echo
